# Bee Well: a positive psychological impact of a pro-environmental intervention on beekeepers’ and their families’ wellbeing

**DOI:** 10.3389/fpsyg.2024.1354408

**Published:** 2024-03-27

**Authors:** Jolanta Burke, Sean Corrigan

**Affiliations:** Royal College of Surgeons in Ireland, Dublin, Ireland

**Keywords:** positive psychology, wellbeing, happiness, flourishing, beekeeping, bees, pro-environmental behavior

## Abstract

Bees are excellent pollinators and serve an essential environmental purpose. However, little is known about the wellbeing impact of bees on humans. This research addressed the knowledge gap concerning the impact of beekeeping on the wellbeing of beekeepers and their families, focusing on the often-overlooked psychological, emotional, and social dimensions. Thirty farmers in Ireland participating in the *Let it Bee* project, aimed at promoting biodiversity and water source protection, were provided with bee hives. Twelve participants were interviewed to explore the effects of beekeeping on their wellbeing. Thematic Analysis was employed to analyze the data, revealing five central themes. (1) The centrality of *pride* in accomplishing environmental and community objectives in the farmers’ beekeeping activities; (2) the evolving sense of *togetherness* with nature, family, and community throughout the project; (3) a profound sense of contributing significantly to the *greater good*; (4) the recognized value of beekeeping for beekeepers’ psychological *growth, flow, and relaxation*; and (5) the opportunity for beekeepers to leverage their *character strengths*. The implications of these findings are discussed within the framework of their impact on environmental conservation, healthcare policymaking, and implications for research and practice.

## Introduction

The beneficial impact of honeybees on human health benefits ranges from disease prevention to therapeutic applications (Lima et al., 2021; Nainu et al., 2021). Additionally, honeybees significantly contribute to the attainment of various United Nations Sustainable Development Goals, particularly in the realms of food security and biodiversity (Patel et al., 2021). Their symbiotic relationship with nature renders them vulnerable to environmental pollutants affecting water, soil, and earth, necessitating global community efforts to safeguard their wellbeing (Smart et al., 2016; de Jongh et al., 2022). In Ireland, public sentiment reflects concerns, with 88% of individuals expressing dissatisfaction with the government’s efforts to preserve bee populations (NBDC, 2018). Despite emerging research highlighting the potential negative impact of managed bees on wild bee populations (Iwasaki and Hogendoorn, 2022), the critical role of bees in fostering positive health outcomes for both humans and the planet remains integral (UNEP, 2022).

In addition to the environmental and human health benefits, beekeeping has recently garnered attention as a potential source of emotional, psychological, and social wellbeing. Case studies examining the lives of beekeepers during the COVID-19 pandemic highlight the positive impact of online social connections among beekeepers, mitigating feelings of loneliness and enhancing social resources during periods of self-isolation (Alton and Ratnieks, 2022). This social aspect is similar to mindfulness practices, offering stress relief, anxiety prevention, and depression mitigation as beekeepers find solace in observing bees at work in their gardens. Moreover, creativity emerges as an outcome of beekeeping, as beekeepers ingeniously utilize bee produce. Although the literature describes various wellbeing behaviors facilitated by beekeeping during the crisis, its primary objective is to assess how beekeeping aids in coping rather than fostering long-term wellbeing.

Despite the increasing recognition of beekeeping’s potential impact on wellbeing, to authors’ knowledge, only two studies have explicitly explored this phenomenon. A survey involving 192 farmers and focus groups with 111 farmers in Vietnam revealed improved family relations and increased community respect following a beekeeping intervention (Yap et al., 2015). Another study surveyed 86 Italian beekeepers and conducted interviews with 23, identifying positive emotions, psychological flow, amazement, and social connection as key outcomes of beekeeping (Whitaker, 2023). Notwithstanding their valuable insights, these studies adopt entomological (the study of insects) and anthropological (the study of the origins of human society and cultural development) perspectives, lacking a dedicated psychological framework. The current research aims to address this gap by providing an in-depth examination of beekeepers’ emotional, psychological, and social experiences, utilizing specific wellbeing frameworks deriving from Positive Psychology (the study of wellbeing processes, outcomes and characteristics) to assess the broader societal impact of beekeeping on wellbeing.

### The current study

The susceptibility of Ireland’s water bodies to pollution primarily emanates from agricultural practices, exemplified by instances such as slurry spillage in rural areas. This phenomenon has led to a catastrophic decline in biodiversity, emerging as the predominant threat to Irish water bodies, leaving only 0.6% of the nation’s rivers classified as pristine (DHPLG, 2018). *The National Biodiversity Plan* highlights Agriculture, Forestry, Fisheries, and habitat loss as the principal threats to EU protected habitats (NPWS, 2017). Adherence to existing environmental regulations alone is insufficient to reverse this concerning trend. Although scientific knowledge has progressed significantly in addressing these challenges, the translation of scientific insights into actionable community and individual measures remains a formidable task. Recognizing this, the *National Federation of Group Water Scheme* initiated the *National Groundwater Source Protection* pilot program in County Roscommon in 2019. This comprehensive initiative encompassed various components, including providing 30 farmers with beehives, training, and mentoring to foster environmentally friendly farming practices and cultivate a heightened appreciation for nature. Other program strands involved initiatives to safeguard water bodies, preserve wild bees, reduce pesticide application, implement nature-based solutions for water body protection, wild bee preservation, and the creation of riparian habitats to enhance biodiversity and nutrient capture.

An illustrative impact example from the program involves a farmer who received beehives. In response, the farmer planted 4,500 native hedge plants and established a native apple orchard on their farm to support the bees. He encouraged neighboring farmers to do the same. Thus, one of them planted 1,000 native hedge plants, while another established a wild meadow covering a 3,600 sq. m area. This cascade of positive changes occurred in response to a farmer receiving beehives. These collective actions had demonstrable positive effects on water quality, biodiversity, and the overall climate within the catchment.

Farmers are often characterized as “accidental environmentalists” due to their engagement in pro-environmental behaviors motivated by various non-ecological factors, including the health and wellbeing of their families (Marr and Howley, 2019). Evidence indicates a correlation between farmers’ symptoms of mental illness, such as depression, distress, and suicidal ideation, and farm-related stressors such as droughts or crop failure (Batterham et al., 2015). Despite this, the concept of wellbeing, construed as the presence of positive psychological resources, has been overlooked in farming and environmental research (Saxby et al., 2018; Burke et al., 2023). Consequently, the current research endeavored to evaluate the impact of the *Let it Bee* biodiversity project on the wellbeing of farmers and their families, recognizing the potential bidirectional relationship between pro-environmental behaviors and wellbeing.

### Positive psychology perspective

Positive psychology (PP) represents a paradigm within the scientific study of human wellbeing that focuses on elucidating what is right with individuals, as opposed to pathology-oriented perspectives (see Burke, 2021 for a review). The conceptual trajectory of inquiry within this domain has undergone a transformation, initially cantered on the examination of positive attributes, processes, and institutions (Seligman and Csikszentmihalyi, 2000). This trajectory has evolved to encompass an acknowledgment of the positive consequences arising from adverse states, and prompting an awareness of the requisite conceptual and methodological intricacies involved in integrating positive psychological perspectives with other realms of research (Lomas and Ivtzan, 2016; Lomas et al., 2021).

This stands in contrast to traditional wellbeing frameworks informed by medical perspectives, which emphasize the absence of diseases or infirmities, such as depression or anxiety (WHO, 2023). Wellbeing, as conceived from the vantage point of positive psychology, delves into emotional, psychological, and social resources. These resources not only act as preventive factors against mental illness and mood disorders but also contribute to the promotion of flourishing and the development of wellbeing resources (Schotanus-Dijkstra et al., 2019). Flourishing, in this context, is defined as the pinnacle of emotional, social, and psychological functioning (Keyes, 2002). The resources constituting wellbeing from the positive psychology perspective serve as the underpinning for fostering psychological resilience and a meaningful life.

Various models and measures of flourishing have been proposed, with the Mental Health Continuum by Keyes (2002) being the most prevalent, exhibiting robust validity and providing substantial data on the impact of flourishing on wellbeing (Iasiello et al., 2022). The Mental Health Continuum encompasses two primary theories of wellbeing: (1) Psychological Wellbeing, drawing on Ryff (1989, 2014) concept of eudaimonia (living a virtuous life), and (2) Subjective Wellbeing, rooted in Diener (2012) concept of Subjective Wellbeing underpinned by hedonism (experiencing more pleasure than pain in life). Additionally, it incorporates a model of Social Wellbeing proposed by Keyes (1998). Recently, an alternative model emphasizing living a psychologically rich life, leading to optimal functioning, has been introduced (Oishi and Westgate, 2022). These theories collectively represent the positive psychological perspective on wellbeing, reflecting a multifaceted approach that encompasses diverse wellbeing components, culminating in the overarching outcome of flourishing.

Despite widespread reports of happiness, only a minority of individuals experience flourishing, and this state offers protection against mood disorders and further symptom development (Schotanus-Dijkstra et al., 2019; Burns et al., 2022). Flourishers are at significantly lower risk of a major depressive episode compared to languishers (individuals reporting low levels of emotional and psychological functioning) and those moderately well (individuals reporting mixed levels of emotional and psychological functioning) (Keyes, 2002). Flourishing, however, exhibits fluidity, as evidenced by a 50% decline in flourishing over a decade, with subsequent implications for mental health outcomes (Keyes et al., 2010). Interventions grounded in the positive psychology framework have the potential to elevate the population’s health by fostering higher levels of optimal functioning, thereby reducing the incidence of mental health issues (Huppert, 2005).

In summary, the Bee Well project sought to examine the wellbeing impact of pro-environmental initiatives by investigating the effects of the *Let It Bee* biodiversity project on farmers and their families. In light of the limited scholarly attention within the realm of positive psychology concerning this subject, the present study aims to make a distinctive contribution to the expanding body of research aimed at improving wellbeing. Furthermore, this contribution extends to the broader field of environmental research, encompassing the exploration of diverse sources and ramifications of pro-environmental interventions, such as wellbeing, with the overarching objective of environmental preservation and a collective commitment to climate protection.

## Method

This qualitative research project was conducted over the period from February to June 2023, following rigorous ethical considerations. Ethical approval was obtained from the RCSI (Royal College of Surgeons in Ireland) ethics committee, aligning with the Charter of Fundamental Rights of the European Union. The research adhered to ethical guidelines outlined in the European Convention on Human Rights and its supplementary Protocols, as well as the Declaration of Helsinki and the European Code of Conduct for Research Integrity. Compliance with the EU General Data Protection Regulation (Regulation (EU) 2016/679) (GDPR) governed the use and transfer of pseudonymized participant data.

### Participants

The study involved a cohort of 12 farmers, comprising ten males and two females. Age distribution included five participants aged 60 and above, four aged 50–59, two aged 40–49, and one participant aged 18–29. In terms of farm size, three participants owned small farms, three assessed their farms as medium-sized, and six managed large farms.

### Procedure

The research design encompassed two key components: (1) a pre-interview survey and (2) one-to-one semi-structured interviews.

### Pre-interview survey

Before the semi-structured interviews, participants completed a brief survey regarding their involvement in the *Let it Bee* project. This survey collected demographic data (age, gender, and farm size) and provided an avenue for participants to share narratives about their engagement in the *Let it Bee* project. Specifically, participants they were asked 5 questions about the project, which they assessed on a 5-point Likert scale ranging from strongly disagree to strongly agree. The questions stated: (1) The *Let it Bee* project made my life (a) more enjoyable, (b) more meaningful, (c) more profitable, (d) more stressful; (2) the project helped me connect with my community; (3) The project has improved the quality of my family’s life; (4) The project has improved biodiversity in my area; (5) The project has improved water quality in my area. The objective of asking these questions was to establish a foundational context for participants, affording them the opportunity to engage in reflection regarding the influence of bees on various aspects of their lives. This preliminary grounding served as a prelude to eliciting participants’ personalized perspectives of the observed wellbeing impact. Descriptive analysis of the survey data was conducted using SPSS (version 27).

### Interviews

In-depth “walking” interviews were employed to explore the impact of the *Let it Bee* project on farmers’ wellbeing and their families. This innovative data collection method involved researchers and participants engaging in conversation while walking together, facilitating the collection of narratives and stories related to the research topic (O’Neill and Finnegan, 2020). This approach aimed to deepen the understanding of farmers’ experiences within their natural habitat, where they typically feel most at ease.

The interview schedule comprised questions relating to (1) participants’ involvement in the *Let it Bee* project (why they decided to do it, how their family reacted to it, what obstacles they have experienced and what were the outcomes of their struggle), (2) the impact that the project had on them, (3) the impact that the project had on their family. The prompts included such questions as whether *Let it Bee* project changed their relationships with people, impacted their priorities in life, changed their health in any way and what attitudes or behaviors it altered (if any).

All interviews were recorded using two smartphone recording applications as a precautionary measure in the event of a technological failure. The interviews were conducted by both authors and subsequently transcribed by one author. The anonymization of all data occurred during the transcription process. Preceding and following each interview, both authors actively participated in reflective practice, using a notebook. Subsequent to each interview session, they dedicated time to articulate and deliberate upon their reflections, encompassing observations, cognitive impressions, preconceptions, and emotional responses evoked during various junctures. At the end of each data-collection day, additional reflective insights were incorporated into their respective reflective diaries, and the data gleaned from their individual notes was referred to at the analytical phase of the research. The rich, detailed, multisensory data obtained from the walking interviews underwent analysis using a combination of inductive and deductive Thematic Analysis (Braun and Clarke, 2021). Following the input of data into the MAXQDA software (2022), the preliminary coding process was undertaken by one researcher, while the subsequent coding and developing initial themes were carried out by the second researcher. This sequence was succeeded by a three-hour meeting during which all identified themes were discussed and mutually agreed upon.

## Results

### Part 1: pre-interview survey

The preliminary phase of the research involved the administration of a survey before conducting interviews, aiming to assess the influence of the *Let it Bee* project on participants’ wellbeing and their perceptions of associated environmental changes. The survey results were categorized into three distinct groups: (1) wellbeing, (2) environment, and (3) financial, as detailed in Table 1.

**Table 1:** Pre-interview survey results (*N* = 12).

Variable	Descriptive data		Frequency data (*n*, percentage)				
	M	SD	Strongly disagree	Disagree	Neither agree nor disagree	Agree	Strongly agree
Wellbeing							
Enjoy	4.2	0.9			4, 33.3	2, 16.7	6, 50
Meaning	4	1		1, 8.3	2, 16.7	5, 41.7	4, 33.3
Connection	4	0.9			4, 33.3	4, 33.3	4, 33.3
Stress	2	1.4	6, 50	2, 16.7	1, 8.3	1, 8.3	1, 8.3
Quality of Life	3.5	0.9		1, 8.3	6, 50	3, 25	2, 16.7
Environment							
Biodiversity	4.2	1.2	1, 8.3		1, 8.3	4, 33.3	6, 50
Water quality	3.8	1.1	1, 8.3		3, 25	5, 41.7	3, 25
Financial							
Profit	2.2	1.5	6, 50	2, 16.7	2, 16.7		2, 16.7

The majority of participants expressed sentiments of enjoyment and meaning derived from beekeeping, viewing it as a means of interpersonal connection. While two participants reported finding beekeeping stressful, the prevailing sentiment among others did not align with this perspective. Interestingly, only five participants acknowledged a positive impact on their overall quality of life. Concerning the environment, a consensus emerged among most participants affirming that beekeeping has contributed to the enhancement of biodiversity in their respective areas, with some noting observable improvements in water quality. It is noteworthy that a majority of participants did not report financial gains from their beekeeping endeavors.

### Part 2: interviews

**Figure 1:**
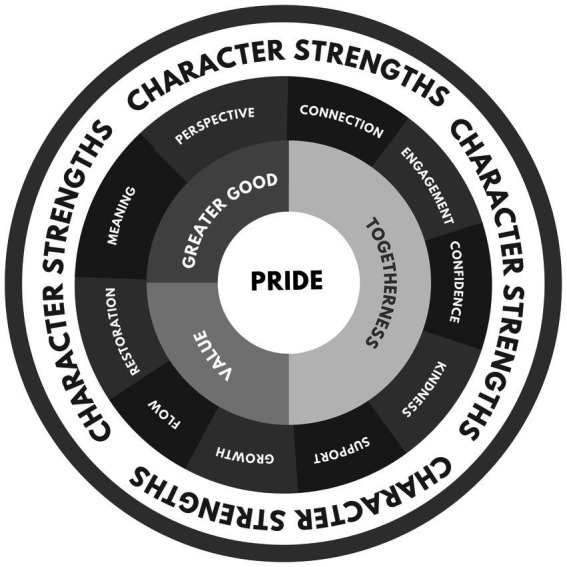
Bee Well research themes.

The qualitative analysis of interview data unveiled five prominent themes: (1) pride, (2) togetherness, (3) greater good, (4) value, (5) character strengths (Table 2 and Figure 1).

**Table 2:** Bee Well research themes.

Theme number	Theme	Subtheme
1	Pride	
2	Togetherness	Connection
		Engagement
		Confidence
		Kindness
		Support
3	Value	Growth
		Flow
		Restoration
4	Greater Good	Meaning
5	Character strengths	Perspective

### Pride

Every participant conveyed a sense of pride intricately connected to their beekeeping endeavors, stemming from diverse sources. Some participants took pride in the quality of their produce.

“*A friend from football called in and she’d get a jar of honey, she’d say it was the best honey and she’d call again for more*” (Sorcha, Pos. 48).

Conversely, others found pride in contributing to community health.

*“Locals have rung me up looking for honey because they had kids in the house who had asthma”* (Walter, Pos. 52).

Participants also expressed pride in the environmental impact of beekeeping activities.

*“There were berries (on the trees). We never had that, we have it now and it’s all down to the bees*” (Oisin, Pos. 92).

Additionally, some participants took pride in the collective efforts of their community, highlighting instances where service users from a local Mental Health hospital contributed to the project by crafting bee-hotels.

*“The people who were making them would say: we have five of them ready when are you taking them. They became very emotional when they were doing it, and it was something different”* (Walter, Pos. 89).

Consequently, the pervasive sentiment of pride resonated throughout all interviews, influencing diverse facets of farmers’ lives.

### Togetherness

The theme of togetherness revolves around beekeepers establishing a sense of unity with their families, community, fellow beekeepers, nature, and the bees. This overarching theme encompassed five distinct subthemes that emerged under the category of togetherness, (1) connection, (2) engagement, (3) confidence, (4) kindness, and (5) support.

#### Connection

Each participant consistently reported experiencing a heightened connection to nature and bees, surpassing previous engagements with the care of cattle or the cultivation of crops. This connection transcended mere observation; participants actively listened to and observed bees, fostering a profound sense of oneness. Some participants detailed a synchronization with bees to the extent that the insects’ emotional states influenced their own; when bees exhibited agitation, participants similarly experienced distress, and conversely, calmness in the bees elicited a corresponding tranquility in the participants. This deepened connection prompted participants to adopt a perspective aligned with that of the bees, fostering a mutual understanding. Here is an example of this bee-human symbiosis resulting from the established connection is provided:

“*You’d look at the hive of bees, at the entrance see what they're doing, and you can tell a lot about the weather”* (Colm, Pos. 21).

Participants also established connections with local beekeepers; consequently, individuals experiencing loneliness connected with others.

“So, you make friends (…). That is probably good for your mental health, a positive thing for a lot of people” (Eamon, Pos. 130).

Selected participants have opted to join beekeepers’ clubs, thereby expanding their social network and discovering a community with whom they could establish deeper connections.

“There are some really lovely people, they don't impose themselves on you, and would help you in the club” (Raymond, Pos. 134).

Beekeepers established connections with strangers who, upon discovering their involvement in beekeeping, stopped their cars to inquire. They also engaged more with neighbors with whom they traditionally exchanged only greetings. Since acquiring bees, neighbors regularly approached them with enquiries about bee-related matters, seeking guidance on farming practices to ensure the wellbeing of the bees. The presence of bees has emerged as a catalyst for initiating conversations, facilitating numerous participants in establishing connections within their community.

“*My neighbor up the road rang me one day, she wanted to go spraying briars and asked me what I think of it, if I didn’t have the beehives, she wouldn’t have rang me”* (Walter, Pos. 48).

Ultimately, participants cultivated deeper connections with their families. Grandparents forged bonds with their grandchildren, who now associated any bee sighting with their grandparents’ beekeeping endeavors. Parents articulated a commitment to enhanced quality time with their children, focusing on tending to the bees and distancing themselves from digital devices. For parents with adult children, the shared challenge of beekeeping provided a new avenue for connection and collaboration within the familial unit.

*“We probably spend more time together, a lot of time harvesting honey*” (Sorcha, Pos. 22).

#### Engagement

Numerous participants emphasized that beekeeping evolved into an engaging communal activity. It transcended mere social connections by fostering shared, profound interest in bees, enabling individuals to elevate their relationships without self-consciousness. Participants dedicated considerable time to discussions and interactions cantered around this shared fascination. An illustrative example involves a childless participant who actively involved his niece in beekeeping, leading to joint activities such as sharing books, viewing bee-related videos, and harvesting honey together. Parents with underage children recounted their children’s heightened engagement, expressing interest in joining beekeepers’ clubs for adults. Beekeepers, irrespective of their typical reading habits, demonstrated an inclination to purchase books as part of their commitment to expanding their knowledge about bees.

*“I got a suit for her when she's coming down during the summer. We spend time looking at them and seeing what they're doing”* (Ciaran, Pos. 37).

#### Confidence

Beekeepers articulated a notable increase in their personal confidence. Specifically, one participant disclosed a history of mental health issues and shared that tending to the bees had significantly enhanced his happiness and confidence, fostering a belief in his ability to contribute meaningfully to both the community and the environment. Other participants highlighted the positive effects of beekeeping on their children, noting an observed increase in their children’s self-assurance. Being recognized as beekeepers at school provided their children with a distinct identity, resulting in frequent requests for them to speak about their beekeeping experiences within the school community.

*“There is a huge change. They were sitting in the classroom, and they couldn’t talk about anything. They are not into football (…) but now they have something to talk about in the class. And they would stand up and everyone would sit and listen, including the teachers. It builds their confidence hugely.”* (Colm, Pos. 46).

#### Kindness

The majority of participants highlighted the benevolence engendered by bees within the community. Beekeepers actively engaged in acts of kindness, exemplified by the distribution of honey jars to their neighbors. This altruistic gesture was rooted in the belief that the bees were communal entities, contributing to the overall wellbeing of the community rather than serving individual beekeepers. Notably, the bees utilized community gardens for pollination, reinforcing the communal aspect of their presence. Additionally, participants extended these acts of kindness by sharing honey with their family members and friends.

*“I wouldn’t put a monetary value on it either. It’s a gift. A gift that you won't get everywhere because it's a pure natural gift”* (Brian, Pos. 54).

Ultimately, a considerable number of beekeepers engaged in reciprocal acts of kindness toward one another. Drawing inspiration from the bees’ model of a harmonious society, they sought to contribute to their human “hive” in a manner analogous to the bees’ communal collaboration. This demonstrated an alignment between the behavior observed in bee societies and the reciprocal kindness practiced within the human community of beekeepers.

*“The gift of nature (...) so that there is a sense of community”* (Bridget, Pos. 88).

#### Support

Participants marveled at the reciprocal support evident within their beekeeping network. This support extended beyond financial transactions, with beekeepers willingly assisting each other in situations where bee-hives faced challenges without expecting remuneration. The support network operated through various channels, including regular interactions during network meetings and communication through phone calls to discuss issues related to bee-hives. Additionally, participants highlighted the sense of support received from the broader community, particularly in the context of environmental initiatives aimed at enhancing the survival of bees. Notably, this support also encompassed the engagement of families fully involved in beekeeping activities.

*“It is nice to impart knowledge to others I wouldn’t be anywhere unless I had good mentors behind me”* (Brendan, Pos. 31).

### Value

The thematic focus on value in the context of beekeeping encapsulated three central themes: (1) growth, (2) flow, and (3) restoration.

#### Growth

The majority of beekeepers expressed their commitment to a lifelong educational journey cantered around the intricate understanding of bees. While the learning trajectory proved steep for some and less challenging for others, all participants acknowledged the significance of continuous learning, fostering the growth of their expertise in beekeeping.

*“It's a skill you can't learn from a book. (...) It was a new thing in the evenings to do. I don't go football and or anything like that it's like another bit of hobby”* (Colm, Pos. 6).

#### Flow

Numerous participants elaborated on their encounters with a state of flow, noting the phenomenon of losing track of time and self-awareness while tending to bees. One farmer disclosed spending entire Saturdays engrossed in beekeeping to the extent of forgetting to eat lunch. Parents shared anecdotes of their children becoming deeply engrossed in beekeeping, leading them to lose themselves in the activity and spend extended periods at the beehive instead of watching the TV or playing online games.

*“All you do is think about the bees, focus on the task*” (Sorcha, Pos. 78).

#### Restoration

The majority of participants discussed the rejuvenating significance of beekeeping, highlighting its capacity to elicit a surge of positive emotions. These emotions encompassed feelings of serenity, calmness, awe, and unadulterated happiness while observing the industrious activities of the bees. One participant articulated the act of turning to beekeeping as an escape during tumultuous periods at home, describing how it facilitated a process of cantering oneself and recuperating from the challenges posed by the demands of daily life.

*“You just go down and take a couple of hours (…) it's a relaxing couple of hours”* (Walter, Pos. 28).

### Greater good

The thematic element of “Greater Good” surfaced consistently throughout all participant interviews, delineating the beekeepers’ capacity to go beyond individual perspectives and recognize the transcendent nature inherent in the practice of beekeeping. This theme comprises two subthemes, (1) meaning, and (2) perspective.

#### Meaning

Participants recognized that beekeeping surpassed a mere leisure pursuit centered on honey production, embodying a profound significance that extended to their ecological impact. Prior to engaging in beekeeping, individuals aspired to make a positive contribution to the environment yet were uncertain about how to do it. Beekeeping allowed them to contribute meaningfully to the environmental protection.

*“The awareness of the environment has come much more to the fore; we have to be more aware of what is happening to the climate anyone that is involved in farming”* (Walter, Pos. 56).

Numerous participants articulated their engagement in beekeeping as a facet of their legacy, aiming to contribute to a more sustainable world for subsequent generations. Their motivation was rooted in a desire for younger individuals to recognize their efforts in environmental preservation. A grandparent expressed a wish for their grandchildren to reflect upon the bees and acknowledge the substantial contribution they made to the bees’ survival over the years.

“*They (the neighbors) are not into bees - they do that (…) for the next persons"* (Colm, Pos. 113).

#### Perspective

Several participants highlighted a transformative shift in their perspectives facilitated by their interaction with bees. This experiential learning from bees encompassed insights into leading a virtuous life, conservation of resources, and fostering a connection with nature. Additionally, participants acknowledged gaining valuable lessons from bees about societal dynamics, leading to a heightened awareness of both commendable practices and areas for improvement in their own actions.

*“They are very fascinating if you look at what they do, they way they organize themselves it’s a bit like there is no one in charge but they are all in charge it is the way that society should work, but it doesn’t”* (Raymond, Pos. 13).

### Character strengths

The overarching theme derived from this study indicated that the care of bees has provided farmers with an opportunity to leverage their individual character strengths, irrespective of what they were. Individuals requiring reconnection with nature found the space to achieve this through beekeeping. Those inclined towards training and educating others utilized their strengths by actively participating in bee clubs and teaching others beekeeping skills. Furthermore, individuals possessing leadership skills had an opportunity to use them by leading the change in their community. Consequently, beekeeping emerged as a facilitator for farmers to realize their optimal potential, experience a sense of accomplishment within the area they were good at and become the most refined version of themselves.

“(In my) trade, the detail is important, you know, when you’re doing something, you’re doing it and you’re not thinking about the next job or something else. (…) When I’m with bees I kind of like to block out everything else that's going on in my mind and sort of concentrate on what I’m doing” (Padraic, Pos. 17).

## Discussion

This study represents a pioneering exploration into the positive psychological implications of beekeeping on the wellbeing of beekeepers and their families. The theme of “Togetherness,” previous research highlighted beekeeping as a source of fostering a happier family connection, emphasizing positive interactions with bees and sharing produce with loved ones (Alton and Ratnieks, 2022; Whitaker, 2023). However, the current research expanded upon these family benefits by highlighting intergenerational interest sparked by bees, uniting parents with children, grandparents with grandchildren, and uncles with nieces. The resulting connections equipped young individuals with beekeeping skills, diverting their attention from digital devices, fostering confidence through participation in beekeeping clubs, and allowing them to share knowledge within their school community, thus contributing to positive youth identity development (Tsang et al., 2012).

Furthermore, this study constituted the initial exploration of a phenomenon termed as “positivity resonance” occurring between humans and animals. Positivity resonance involves the co-experience of positive emotions among humans, encompassing non-verbal and biological synchrony (Wells et al., 2022). Preliminary investigations propose that this process correlated with reductions in depressive symptoms, loneliness, and illness-related symptoms, along with enhancements in psychological flourishing (Major et al., 2018). Additionally, it fosters pro-social behavior (Zhou et al., 2022) and contributes to a heightened sense of life meaning (Prinzing et al., 2023). The present study, uniquely, identified a comparable psychological manifestation of positivity resonance between humans and bees, when farmers reflected on how the bees’ emotional experience influenced theirs and vice versa. Further enquiry is imperative to delve into this phenomenon at a biological level, clarifying the similarities and distinctions in wellbeing outcomes associated with positivity resonance between humans and between humans and bees. This discovery may pave the way for future research endeavors investigating positivity resonance between humans and the broader animal kingdom as a mechanism creating the impact of animals and insects on human wellbeing.

In alignment with previous research demonstrating reciprocity as an outcome of beekeeping (Whitaker, 2023), this study revealed community support for participants, transcending mere reciprocity and family support. The evidence suggested that beekeepers felt fully supported by the community in ensuring the survival of their bees. Neighbors actively changed farming practices, cultivated meadows for the bees, erected fences supporting the project, and planted apple orchards. This community support extended beyond expected reciprocation, prompting further investigation into the factors influencing this phenomenon in relation to bees and the potential role of other project- or community-related factors.

The current research expands on the notion of reciprocity by introducing kindness as a prominent theme. Participants perceived bees not as personal possessions but as communal entities, prompting acts of kindness such as gifting neighbor’s jars of honey or providing natural medicine to community members with ailments. The participants’ high valuation of their unique produce accentuated the significance of the positive impact derived from their acts of kindness. Drawing on a systematic review indicating positive wellbeing outcomes for the giver (Curry et al., 2018) and stress reduction associated with acts of kindness (Fryburg, 2021), it is plausible that participants’ lower stress levels in beekeeping are linked to the positive effects of sharing honey.

Regarding the theme of “Value,” the current research aligns with previous studies demonstrating psychological growth, flow, and restoration as outcomes of beekeeping (Yap et al., 2015; Whitaker, 2023). A noteworthy distinction arises between psychological flow and restoration, with the former occurring during challenging situations where individuals can employ their skills. Conversely, restoration involves a state of relaxation allowing for attention deactivation and recovery, such as observing bees. Both states contribute differently to wellbeing outcomes and need to be further researched in the context of beekeeping.

A novel contribution of the present study involves the “Greater Good” theme, specifically the perspective-changing aspect resembling psychological richness (Oishi and Westgate, 2022). Participants, influenced by observing bees and their ideal society, exhibited altered behavior and perspectives towards others. This psychological richness, usually associated with experiences like travel, club memberships, or sports (Oishi et al., 2021), is now extended to beekeeping. Further research is essential to compare the impact of beekeeping on psychological richness with other activities, particularly in the context of environmental sciences, given its established link with pro-environmental behaviors (Wei et al., 2023).

This study the first to demonstrate how beekeeping enables participants to leverage their character strengths, a factor associated with higher reported levels of wellbeing (Lee et al., 2020). Beekeeping facilitated the effective use of strengths, whether through leading change, teaching beekeeping, or expressing kindness when sharing bee produce. This demonstrated the diverse ways in which beekeeping positively impacted individuals based on their character strengths. Given that strength use is associated with psychological flourishing (Hone et al., 2015), an ability for farmers to engage their strengths through beekeeping can become a source of improved wellbeing.

The current study represented a pioneering effort to examine the influence of beekeeping on key facets of wellbeing within the frameworks of flourishing, as conceptualized by Positive Psychology research. Notably, the elements corresponding to PERMA (Seligman, 2011), the Mental Health Continuum (Keyes, 2002), and Psychological Richness (Oishi and Westgate, 2022) were evident in the beekeeping experiences of farmers. Over the years, a range of Positive Psychology Interventions have been created to enhance these dimensions of wellbeing (e.g., Pawelski, 2020; Burke et al., 2023). The results of this study showed that beekeeping has the potential to emerge as a Positive Psychology Intervention, thereby assisting individuals and their families in reinforcing psychological, emotional, and social well-being while concurrently contributing to environmental improvement.

### Implications for policy and practice

The present study represents a significant contribution to the field of climate and environmental science, particularly in clarifying the impact of pro-environmental behaviors such as beekeeping on beekeepers’ and their family’s wellbeing. This contribution holds the potential for changing the conceptual framework for integrated land and landscape management, as envisioned by the Irish-government-funded initiative, An Foram Uisce (2021). The change could relate to the powerful impact of wellbeing as an outcome of not only beekeeping but potentially other pro-environmental actions, which need to be considered alongside environmental research findings.

In contrast to the prevailing perspective that predominantly emphasizes the personal burdens and anxieties associated with environmental protection (e.g., eco-anxiety), the current research diverges by accentuating the personal advantages individuals accrue through active participation in pro-environmental programs. Consequently, these findings offer a novel perspective that could serve as a motivational lever for individuals inclined toward pro-environmental actions, emphasizing the personal benefits associated with engaging in climate-related initiatives.

### Limitations

In spite of its manifold advantages, the extant research exhibits three principal limitations. Primarily, the non-generalizability of the sample necessitates future investigations encompassing a more expansive cohort of beekeepers to corroborate the present findings. Secondly, the *Let it Bee* initiative was designed to incentivize farmers to enhance water quality in the region. This clear objective may have potentially magnified the eudaimonic outcomes of the study for all participants. Subsequent research endeavors should consider the wellbeing impact of beekeepers with other motivations for engagement. Lastly, the current enquiry exclusively gauged the impact on family wellbeing from the vantage point of beekeepers, prompting a recommendation for future investigations to scrutinize the outlooks of individual family members.

In conclusion, this study is the first to explore into the wellbeing ramifications of beekeeping from a positive psychological standpoint. The innovative findings contribute to environmental research by clarifying the interconnections between pro-environmental activities and emotional, social, and psychological wellbeing. Moreover, these findings establish a foundation for further examination of the potential role of beekeeping in improving the wellbeing of individuals and their respective families.

## Data availability statement

The original contributions presented in the study are included in the article/supplementary materials, further inquiries can be directed to the corresponding author.

## Ethics statement

The studies involving humans were approved by Ethics Committee, Royal College of Surgeons in Ireland. The studies were conducted in accordance with the local legislation and institutional requirements. The participants provided their written informed consent to participate in this study.

## Author contributions

JB: Conceptualization, Data curation, Formal analysis, Funding acquisition, Investigation, Methodology, Supervision, Writing – original draft, Writing – review & editing. SC: Formal analysis, Project administration, Resources, Writing – review & editing.
